# Albumin Nanoparticles Harness Activated Neutrophils to Cross Vascular Barriers for Targeted Subcutaneous and Orthotopic Colon Cancer Therapy

**DOI:** 10.3390/jfb17010036

**Published:** 2026-01-10

**Authors:** Zhifan Luo, Liuqing Dong, Yujie Zhang, Mingzhen Zhang

**Affiliations:** School of Basic Medical Sciences, Xi’an Jiaotong University, Xi’an 710061, China; zluo18@alumni.jh.edu (Z.L.); dongliuqing@stu.xjtu.edu.cn (L.D.); zhangyujie@xjtu.edu.cn (Y.Z.)

**Keywords:** albumin nanoparticles, drug delivery system, colon cancer, 6-shogaol

## Abstract

Colorectal cancer (CRC) therapy faces challenges due to limited drug penetration across the blood–tumor barrier. Neutrophils, with their natural ability to migrate to inflamed and tumor sites, offer a promising cell-mediated delivery strategy. This study developed albumin nanoparticles loaded with 6-shogaol (NPs/6-shogaol) and utilized activated neutrophils as carriers to transport the nanoparticles across vascular barriers for colon cancer therapy. The physicochemical properties, biocompatibility, and targeting efficiency of the NPs were evaluated in vitro and in vivo. The formulated NPs/6-shogaol exhibited favorable physicochemical properties, including a uniform nano-scale size (~150 nm), negative zeta potential, and high drug loading efficiency. In both subcutaneous and orthotopic colon cancer models, neutrophil-mediated delivery significantly enhanced tumor accumulation of 6-shogaol, inhibited tumor growth, and induced apoptosis by suppressing neutrophil elastase (NE) expression. Notably, no significant systemic toxicity was observed. This neutrophil-hitchhiking albumin nanoplatform provides a targeted and biocompatible strategy for effective colon cancer therapy.

## 1. Introduction

Colorectal cancer (CRC) remains a globally prevalent malignancy, ranking as the fourth leading cause of cancer-related mortality, with a concerning rise in incidence among younger populations [1,2,3]. In the United States alone, the American Cancer Society estimates approximately 150,000 new diagnoses annually, with about 35% of patients succumbing to the disease [4]. The primary objective of CRC treatment is to achieve complete eradication of cancer cells while preserving healthy tissues, with therapeutic strategies tailored to the patient’s overall condition, tumor location, and disease stage [5]. Current clinical interventions include surgery, chemotherapy, radiotherapy, and targeted therapies [6]. Among these, localized delivery of chemotherapeutic agents holds promise for enhancing efficacy and minimizing systemic side effects. However, effective drug penetration remains hampered by physiological barriers, particularly the blood-tumor barrier (BTB) [7,8].

To overcome this challenge, nanoparticle-based drug delivery systems (NDDS) have been extensively explored, leveraging active targeting ligands or passive accumulation via the enhanced permeability and retention (EPR) effect. Despite these advances, the therapeutic potential of NDDS is often limited by short blood circulation half-lives, inadequate intra-tumoral drug accumulation, and off-target toxicity [9,10].

In recent years, immune cells have emerged as attractive living carriers for drug delivery due to their innate ability to traverse the vasculature, home to sites of inflammation or malignancy, evade immune clearance, and exhibit extended circulation kinetics [11]. Neutrophils, the most abundant leukocytes in peripheral blood, serve as the body’s first line of defense, rapidly infiltrating tissues in response to injury or infection [12]. Their inherent tropism toward inflamed and tumorigenic microenvironments positions them as promising vehicles for targeted delivery of chemotherapeutics and tumor microenvironment modulators [13,14]. Notably, activated neutrophils exhibit a high affinity for albumin nanoparticles, suggesting their potential to actively ferry nanotherapeutics across vascular barriers, thereby improving drug delivery to pathological sites [15,16].

Neutrophil elastase (NE), a serine protease released by neutrophils, has been implicated in cancer progression, promoting tumor invasion and metastasis [17]. In CRC, peritumoral regions often show dense neutrophil infiltration coupled with elevated NE expression [18]. Similarly, in lung cancers, serum NE levels correlate with disease severity and progression [19]. Proposed mechanisms underpinning the pro-tumorigenic role of NE include activation of MAPK signaling, suppression of tumor suppressor pathways, and facilitation of metastatic dissemination [20,21]. These findings highlight NE as a clinically relevant target for therapeutic intervention in neutrophil-rich tumors.

Ginger (*Zingiber officinale*), widely recognized for its culinary and medicinal uses, has been traditionally employed in Asian medicine to treat inflammatory and gastrointestinal disorders [22]. 6-Shogaol, a phenolic constituent of ginger characterized by an α,β-unsaturated ketone group, has demonstrated broad anticancer properties across multiple models, including colon, breast, and lung cancers [23]. Its mechanisms encompass inhibition of proliferation, invasion, angiogenesis, and metastasis, often mediated through induction of apoptosis via reactive oxygen species generation and caspase activation [24]. Additionally, 6-shogaol exhibits notable anti-inflammatory activity by suppressing key mediators such as inducible nitric oxide synthase and cyclooxygenase-2 [25].

**Figure 1:**
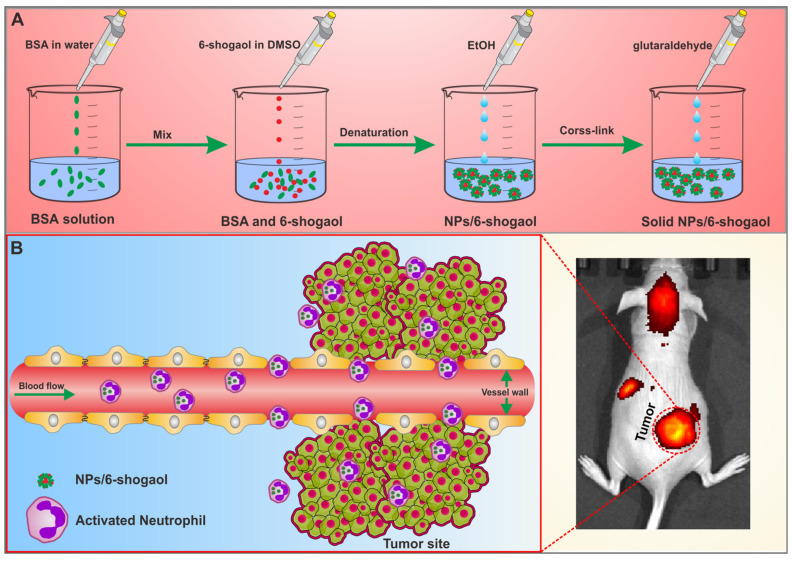
**Illustration of the self-assembled 6-shogaol-loaded albumin NPs (NPs/6-shogaol) for neutrophil-mediated colon cancer therapy.** (**A**) Bull Serum Albumin (BSA) NPs loaded with 6-shogaol were prepared via a green synthesis method. (**B**) Neutrophils mediated the delivery of albumin NPs across the blood vessel barrier for colon cancer therapy.

In this study, we developed albumin nanoparticles loaded with 6-shogaol (NPs/6-shogaol) to exploit the neutrophil transmigration pathway for targeted delivery across vascular barriers in both subcutaneous and orthotopic models of colon cancer (Figure 1). The resulting NPs/6-shogaol displayed a uniform nanosize of approximately 150 nm and a negative zeta potential around –50 mV. By hitchhiking on activated neutrophils, these nanoparticles achieved significant antitumor efficacy in vivo. Mechanistically, 6-shogaol was found to suppress neutrophil elastase expression within tumor tissue. Importantly, the platform showed no appreciable systemic toxicity, offering a biocompatible and targeted therapeutic strategy that integrates the advantages of cellular carriers and nanotechnology.

## 2. Materials and Methods

### 2.1. Preparation of Albumin Nanoparticles Loaded with a Fluorescent Dye (DiL) or 6-Shogaol

BSA nanoparticles (NPs) were prepared using a desolvation method. Briefly, BSA was dissolved in deionized water at a concentration of 20 mg/mL. For plain NPs, 2 mL of the BSA solution was stirred at 1000 rpm, and 7 mL of ethanol was added continuously over 15 min at room temperature. For drug-loaded NPs, DiL (20 mg/mL, 200 µL) or 6-shogaol (20 mg/mL, 200 µL) in DMSO was first mixed with 2 mL of the BSA solution and stirred (1000 rpm) for 15 min. Subsequently, 7 mL of ethanol was added continuously under stirring. To stabilize the NPs, 160 µL of 2% glutaraldehyde was added one hour later to crosslink the BSA molecules. The suspension was then stirred for 5 h at room temperature, followed by centrifugation at 15,000× *g* for 30 min at 4 °C. The NP pellet was washed three times to remove residual DMSO and unencapsulated compounds. Finally, the NPs/DiL and NPs/6-shogaol were resuspended in 5% glucose solution for further use. To quantify the 6-shogaol content, 10 mg of NPs/6-shogaol was dissolved in 1 mL of methanol. After vortexing, the mixture was centrifuged at 5000× *g* for 5 min. A 20 µL aliquot of the supernatant was analyzed using a Shimadzu HPLC system equipped with a UV detector (set at 282 nm) and a reverse-phase C-18 column (5 µm, 10 mm × 150 mm). The mobile phase consisted of 40% H_2_O and 60% acetonitrile for 30 min, followed by 100% acetonitrile. A standard curve was prepared using pure 6-shogaol following the same protocol.

### 2.2. Characterizations of NPs and NPs/6-Shogaol

The hydrodynamic size and zeta potential of both empty nanoparticles (NPs) and 6-shogaol-loaded NPs were determined via dynamic light scattering (DLS) using a Zetasizer Nano ZS (Malvern, Southborough, MA, USA), the concentration of NPs was of 0.5 mg/mL. The measurements were performed at 25.0 °C. Samples were diluted by PBS (pH 7.4). Each sample (n = 3) was measured in a disposable microcuvette with 15 runs of 10 s per measurement. The intensity-weighted harmonic mean (Z-average) diameter and polydispersity index (PDI) are reported. Data were processed using the instrument’s default general purpose analysis model. Atomic force microscopy (AFM) images were acquired with a SPA-400 AFM system (Seiko Instruments Inc., Chiba, Japan). For transmission electron microscopy (TEM) imaging, a droplet of each sample was placed onto a formvar-coated copper grid and allowed to dry at room temperature prior to observation.

### 2.3. Cell Culture

Caco-2, HT-29, and Colon-26 cell lines were grown to confluency in 75-cm^2^ flasks at 37 °C under a humidified atmosphere with 5% CO_2_. HT-29 and Caco-2 cells were maintained in Dulbecco’s Modified Eagle Medium (DMEM), while Colon-26 cells were cultured in RPMI-1640 medium (Life Technologies, Grand Island, NY, USA). All media were supplemented with 100 U/mL penicillin, 100 U/mL streptomycin, and 10% heat-inactivated fetal bovine serum (Atlanta Biologicals, Flowery Branch, GA, USA). All cell lines were obtained directly from the American Type Culture Collection (ATCC), where they had been authenticated via morphology and PCR analysis to exclude interspecies and intraspecies contamination.

### 2.4. Biocompatibility Assay In Vitro

Cell viability of Colon-26 and HT-29 cells following NP exposure was first assessed by MTT assay. Cells were seeded in 96-well plates at a density of 1 × 10^4^ cells per well and incubated overnight. Subsequently, cells were treated with various concentrations of NPs (0, 20, 50, 100, 200, and 500 µg/mL) for 24 and 48 h. After removal of NP-containing medium and one wash with PBS, 20 µL of MTT solution (5 mg/mL) was added to each well, followed by incubation at 37 °C for 4 h until purple formazan crystals were visible. The supernatant was then discarded, and 50 µL of dimethyl sulfoxide (DMSO) was added to dissolve the formazan crystals. Absorbance was measured spectrophotometrically at 570 nm. Untreated cells served as the negative control.

Cellular viability was also evaluated using the ATPLite assay. Colon-26 and HT-29 cells were seeded as described above and exposed to the same NP concentration range for 24 and 48 h. The percentage of viable cells was quantified according to the manufacturer’s instructions. All assays were performed in triplicate.

Real-time cytotoxicity was monitored using electrical cell–substrate impedance sensing (ECIS; Applied BioPhysics, Troy, NY, USA). This noninvasive technique measures changes in AC impedance caused by cell attachment and spreading on electrode surfaces, providing dynamic morphological and viability information. Experiments were conducted using ECIS 8W1E plates, each well containing a small active electrode and a large counter electrode. Caco2-BBE cells were seeded at 2 × 10^5^ cells per well and grown to confluence. Cells were then treated with NPs at concentrations of 0, 20, 50, 100, 200, and 500 µg/mL. Basal impedance was continuously recorded at a frequency of 500 Hz and an applied voltage of 1 V, optimal for Caco2-BBE cells. System control and data acquisition were managed via a lock-in amplifier and dedicated software.

### 2.5. Cell Apoptosis Study

The extent of in vitro apoptosis was evaluated using Annexin V-FITC/propidium iodide (PI) double-staining. Colon-26 and HT-29 cells were seeded onto eight-chamber slides (Tissue-Tek, Sakura, Torrance, CA, USA) and incubated overnight at 37 °C. Cells were then treated with free 6-shogaol (5 µg/mL) or 6-shogaol-loaded NPs (containing an equivalent of 5 µg/mL free 6-shogaol). After 8 h, cells were harvested, washed with ice-cold PBS, and resuspended in 500 µL of Annexin V binding buffer containing 5 µL Annexin V-FITC and 5 µL PI. Following incubation in the dark at room temperature for 15 min, nuclei were counterstained with DAPI. Samples were then observed under a fluorescence microscope.

For quantitative analysis, similarly treated cells were stained with Annexin V-FITC and PI and analyzed using a FACS Canto flow cytometer (BD Biosciences, San Diego, CA, USA). Cell populations were classified as follows: viable cells (Annexin V^−^/PI^−^), early apoptotic cells (Annexin V^+^/PI^−^), necrotic cells (Annexin V^−^/PI^+^), and late apoptotic cells (Annexin V^+^/PI^+^). Excitation was set at 488 nm; fluorescence emissions were collected at 525 nm for Annexin V-FITC and 575 nm for PI. All experiments were performed in triplicate.

### 2.6. Biocompatibility of NPs In Vivo

To evaluate the in vivo biocompatibility of nanoparticles (NPs), FVB/NJ mice (6–8 weeks old) were obtained from Jackson Laboratories (Bar Harbor, ME, USA). Animals were maintained under specific pathogen-free conditions. The mice were randomly divided into 2 groups (n = 3). One group of mice was administered with 200 µL of NPs (5 mg/mL) via intravenous injection once daily for 7 consecutive days. Another group of mice as control was administered with some amount of PBS. Twenty-four hours after the final injection, serum was collected for quantification of pro-inflammatory cytokines (TNF-α, IL-1β, and IL-6) using DuoSet ELISA kits (R&D Systems, Minneapolis, MN, USA). Serum levels of AST and ALT were also measured using corresponding activity assay kits to evaluate potential hepatotoxicity. Body and spleen weights were recorded at the same timepoint. Additionally, hematoxylin and eosin (H&E) staining was performed on paraffin-embedded sections of the heart, liver, spleen, lung, and kidney following standard protocols.

A hemolysis assay was conducted to assess erythrocyte compatibility. Fresh mouse blood was collected into a BD Microtainer MAP tube containing K_2_EDTA (1 mg). Red blood cells (RBCs) were then isolated, purified, and diluted to a concentration of 1 × 10^8^ cells/mL according to a previously described method [26]. Different concentrations of NPs (0, 20, 50, 100, 200, and 500 µg/mL) were incubated with RBC suspensions at 37 °C for 4 h. After incubation, the supernatant from each sample was collected, and absorbance was measured at 540 nm using a microplate reader. A sample treated with 1% Triton X-100 served as the positive control, representing 100% hemoglobin release.

### 2.7. 6-Shogaol and Neutrophil Elastase (NE) to Cell Viability Assay

The effects of 6-shogaol and neutrophil elastase (NE) on cell viability were assessed using the MTT assay. HT-29 cells were seeded in 96-well plates at a density of 1 × 10^4^ cells per well and cultured for 24 h. Cells were then treated with NE (10, 20, 40, 80, 160, and 320 nM), 6-shogaol (10, 20, 40, 80, 160, and 320 µM), or a combination of NE (40 nM) and 6-shogaol (40 µM) for 24 h. After treatment, cell viability was measured following the standard MTT protocol [27].

### 2.8. Mice

Athymic BALB/c nu/nu, C57BL/6, and FVB/NJ mice (6–8 weeks old) were obtained from Jackson Laboratories (Bar Harbor, ME, USA). Animals were maintained under specific pathogen-free conditions. All experimental procedures involving mice were approved by the Institutional Animal Care and Use Committee (IACUC) of Xi’an Jiaotong University (Xi’an, Shaanxi, China).

### 2.9. HT-29 Xenograft Tumor Model

Female athymic BALB/c nu/nu mice were subcutaneously implanted in the right flank with 1 × 10^6^ HT-29 cells suspended in Matrigel (BD Biosciences; diluted 1:1). Once tumor volumes reached approximately 100 mm^3^, mice were randomly allocated into four treatment groups: (1) saline control, (2) free 6-shogaol, (3) blank nanoparticles (NPs), and (4) 6-shogaol-loaded NPs (NPs/6-shogaol). Treatments were administered every three days for a total of 24 days. Tumor volumes and body weights were monitored and recorded at the endpoint. Tumor volume was calculated using the formula: volume = 1/2 LW^2^, where L represents the longest diameter and W the shortest diameter. Animals showing signs of distress, including labored breathing, anorexia, lethargy, or abnormal posture, were humanely euthanized.

### 2.10. Orthotopic Colon Cancer Model

A colitis-associated orthotopic colon cancer model was established based on a previously reported method with minor modifications [28]. Briefly, mice received an intraperitoneal injection of azoxymethane (AOM) at a dose of 10 mg/kg body weight and were maintained on standard diet and water for 7 days. This was followed by two cycles of dextran sodium sulfate (DSS) treatment, each consisting of 2% DSS in drinking water for 7 days, with a 14-day recovery period on regular water between cycles. At the endpoint, colonic tumors were enumerated and measured under a dissecting microscope.

### 2.11. Histological and TUNEL Examination

For histological examination, tumor tissues and major organs (heart, liver, spleen, lung, and kidney) were fixed in 10% neutral buffered formalin for 48 h and subsequently embedded in paraffin. Sections (5 μm) were deparaffinized and stained with hematoxylin and eosin (H&E). Imaging was performed using an Olympus microscope equipped with a Hamamatsu DP-26 digital camera. Apoptosis in tumor tissues was assessed via 4′,6-diamidino-2-phenylindole (DAPI) staining combined with terminal deoxynucleotidyl transferase dUTP nick-end labeling (TUNEL) assay, following the manufacturer’s protocol for a commercial apoptosis detection kit. Images were acquired using an Olympus microscope fitted with a Hamamatsu ORCA-03G digital camera.

### 2.12. Neutrophil Elastase Detection and Imaging

For neutrophil elastase (NE) detection in HT-29 xenograft tumors, frozen tissue sections (6 μm thick) were prepared using a cryostat microtome. The presence of NE protein was visualized using the Naphthol AS-D Chloroacetate Esterase (NAS-DCE) staining method, performed in accordance with the manufacturer’s protocol.

NE activity was monitored in vivo using the neutrophil elastase-activatable fluorescent probe Neutrophil Elastase 680 FAST (PerkinElmer, Waltham, MA, USA). This agent is optically silent upon injection and generates near-infrared fluorescence (680 nm) upon cleavage by neutrophil-derived elastase. HT-29 xenograft-bearing mice were injected intravenously via the tail vein with 4 nmol of the probe in 100 μL. After 4 h, whole-body fluorescence imaging was performed using an IVIS^®^ Series preclinical in vivo imaging system (PerkinElmer, Waltham, MA, USA). Fluorescence intensity in tumor tissues and major organs was quantified and compared.

### 2.13. Immunofluorescence Staining

Tissue specimens were first blocked with 10% normal serum (from the same species as the secondary antibody host) in PBS for 20 min to reduce nonspecific binding, followed by washing with PBS. Next, samples were incubated with primary antibody (5 µg/mL in PBS containing 1.5% normal serum) for 60 min, then washed three times with PBS (5 min per wash). Subsequently, specimens were incubated with Alexa Fluor^®^ 488-conjugated secondary antibody (diluted 1:200) for 1 h, followed by three PBS washes. Finally, coverslips were mounted using DAPI-containing medium and examined under a fluorescence microscope with appropriate filter sets.

### 2.14. Neutrophils Containing NPs/DiL in Blood

Neutrophils were isolated from whole blood using Pluriselect anti-mouse Ly6G S-protein beads (Pluriselect, Spring Valley, CA, USA), following the manufacturer’s instructions. Isolated neutrophils were washed three times with 3 mL of HBSS containing 5% BSA (centrifugation: 300 g, 10 min per wash) and finally resuspended in HBSS supplemented with 5% BSA. For fluorescence imaging, neutrophils were labeled with anti-Ly-6G/Ly-6C antibody conjugated to a fluorophore.

### 2.15. Statistical Analysis

Data are expressed as mean ± standard deviation (SD). Statistical significance was assessed using one-way or two-way analysis of variance (ANOVA), followed by appropriate post hoc tests, or unpaired Student’s *t*-test where applicable. Significance levels are indicated as follows: * *p* < 0.05, ** *p* < 0.01, *** *p* < 0.001.

## 3. Results

### 3.1. Characterization of Albumin NPs and the Targeting Ability to Neutrophils

**Figure 2:**
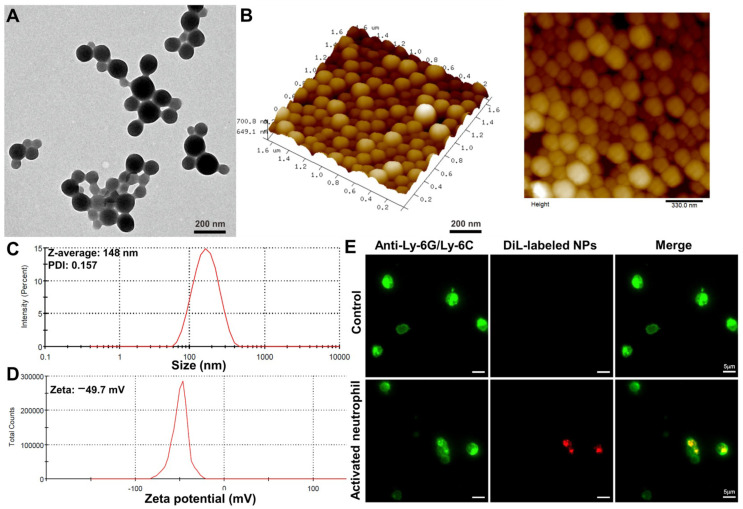
**Characterization of albumin NPs and the targeting ability to neutrophils.** (**A**) Representative Transmission Electron Microscope (TEM) image of albumin NPs. (**B**) Representative Atomic Force Microscope (AFM) image of albumin NPs. (**C**,**D**) Particle size and zeta potential of albumin NPs were characterized by dynamic light scattering (DLS) (n = 3). (**E**) Fluorescence imaging of activated neutrophils (Alexa Fluor^®^488 anti-mouse Ly-6G/Ly-6C (Gr-1) antibody) (Green channel) from tumor-bearing mice after administration of DiL-labeled albumin NPs (Red channel). Scale bar: 5 μm.

Transmission Electron Microscopy (TEM) results of both albumin nanoparticles and the NPs/6-Shogaol formulation (formed by loading 6-Shogaol into the nanoparticles) showed that both structures exhibited a spherical morphology. The albumin nanoparticles possessed an average diameter of approximately 80 nm, whereas the NPs/6-Shogaol displayed an average diameter of approximately 100 nm (Figure 2A and Figure S1A). Atomic Force Microscopy (AFM) characterization further confirmed the spherical morphology of both samples, showing smooth surfaces. Height measurements indicated an average height of approximately 60 nm for the albumin nanoparticles and approximately 90 nm for the NPs/6-Shogaol (Figure 2B and Figure S1B). Hydrodynamic diameter analysis via Dynamic Light Scattering (DLS) showed that the albumin NPs had a hydrodynamic diameter of ~148 nm with a polydispersity index (PDI) of 0.157, and the zeta potential was −49.7 mV, demonstrating excellent dispersibility (Figure 2C,D). The NPs/6-Shogaol exhibited a hydrodynamic diameter of ~145 nm and a PDI of 0.114, and the zeta potential was −50.8 mV (Figure S1C,D). Finally, High-Performance Liquid Chromatography (HPLC) analysis confirmed the successful loading of 6-Shogaol into the albumin NPs (Figure S2). Fluorescence imaging of activated neutrophils (Alexa Fluor^®^488 anti-mouse Ly-6G/Ly-6C (Gr-1) antibody) (Green channel) from tumor-bearing mice after administration of DiL-labeled albumin NPs (Red channel) showed that there was significant overlay fluorescence, indicating the targeting ability of albumin NPs to neutrophils (Figure 2D).

### 3.2. The Biocompatibility of Albumin NPs Against Colon-26 and HT-29 Cells Was Evaluated In Vitro

**Figure 3:**
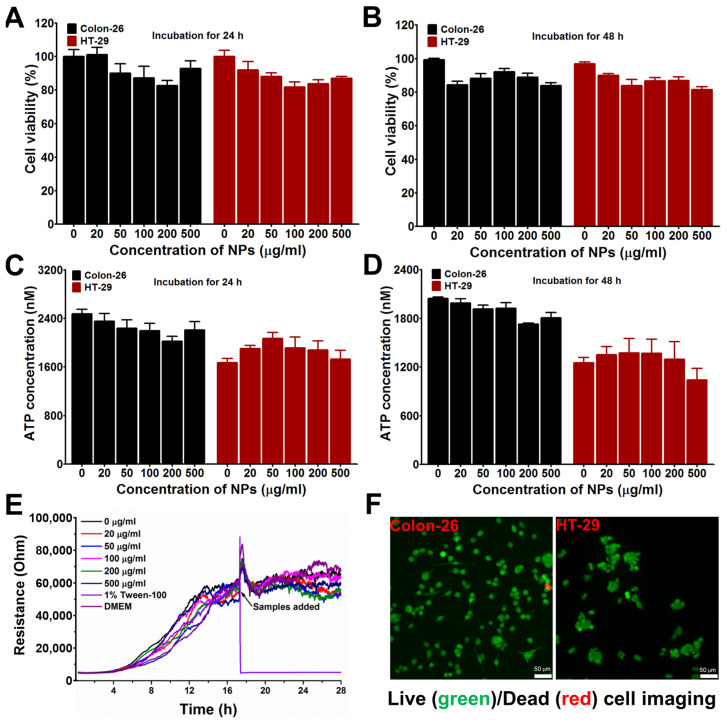
**The in vitro biocompatibility of albumin nanoparticles (NPs) was evaluated in Colon-26 and HT-29 cell lines.** (**A**,**B**) Cell viability was assessed via MTT assay (n = 5). (**C**,**D**) Cell proliferation was measured using the ATPlite assay (n = 5). (**E**) Real-time cell proliferation was monitored with electrical cell–substrate impedance sensing (ECIS) (n = 5). (**F**) Cell viability was further examined by live/dead staining (live cells: green; dead cells: red). Scale bar: 50 μm.

Firstly, the biosafety of the nanoparticles was tested. The concentration of nanoparticles from low to high (20–500 µg/mL) had little effect on the survival of both Colon-26 and HT-29 cells, whether incubated for 24 or 48 h (Figure 3A,B). ATP concentration is one of the gold standards for determining whether the cell is alive and healthy. The ATP level in dead or dying cells drops sharply. The results showed that the concentration of nanoparticles from low to high (20–500 µg/mL) had little effect on the ATP concentration of both Colon-26 and HT-29 cells, whether incubated for 24 or 48 h, which indicated the NPs had good biological safety (Figure 3C,D). Electric cell–substrate Impedance Sensing (ECIS) technology realizes label-free and non-invasive dynamic analysis of cell behavior by monitoring the impedance changes on the electrode surface caused by cell attachment, proliferation, or migration in real time. When cells grow on the electrode, they develop an impedance to alternating current. As the number of cells increased and the area covered by the electrode increased, the resistance value increased accordingly. Therefore, the proliferation of cells can be reflected in real time by monitoring the changes in electrical resistance over time. The ECIS results showed that the resistance of the cells was maintained at a stable level after adding the samples, indicating NPs had no influence on the proliferation of cells (Figure 3E). AM (Calcein-AM)/PI (propidium iodide) staining is a commonly used method to measure cell viability. AM is a non-fluorescent dye that penetrates the cell membrane of living cells and is hydrolyzed to Calcein by esterase inside the cell, which emits green fluorescence. PI, on the other hand, cannot penetrate the intact living cell membrane, but can only enter the nucleus of dead or apoptotic cells and bind to DNA to produce red fluorescence. With this staining method, live and dead cells can be effectively distinguished. The results showed that after incubation with NPs, both Colon-26 cells and HT-29 cells showed green fluorescence, indicating that there was little cell death and the NPs were almost non-toxic to cells (Figure 3F). The potential of the nanoparticles to induce apoptosis was assessed using the Annexin V-FITC/PI staining assay. The results demonstrated that no apoptosis was induced in either HT-29 cells or Colom-26 cells, even at nanoparticle concentrations as high as 500 μg/mL, which further supports the cellular biocompatibility of the nanoparticles (Figure S3). In conclusion, the NPs did not affect either cell proliferation or cell viability, indicating NPs had good biocompatibility.

### 3.3. The Biocompatibility of Albumin NPs Was Evaluated In Vivo

**Figure 4:**
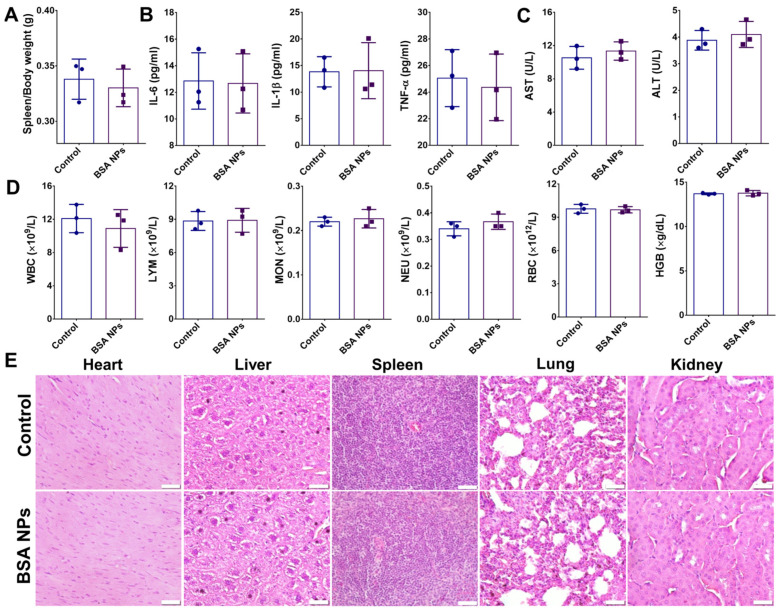
**The biocompatibility of albumin NPs was evaluated in vivo.** (**A**) Spleen/Body weight. (**B**) Anti-inflammatory cytokines (IL-6, IL-1β, and TNF-α). (**C**) Alanine aminotransferase (ALT) and aspartate aminotransferase (AST). (**D**) Hematology parameters (WBC, LYM, MON, NEU, RBC, and HGB). (**E**) H&E staining of vital organ tissues. Representative images are shown (n = 3). Scale bar: 20 µm.

Next, the biocompatibility of albumin NPs in vivo was investigated. Before conducting in vivo safety assessments, the hemocompatibility of the nanoparticles was evaluated first (Figure S4A). The results of the hemolysis assay demonstrated that red blood cells did not exhibit hemolysis even at nanoparticle concentrations as high as 1000 μg/mL, confirming the excellent hemocompatibility of these nanoparticles (Figure S4B). Seven days after the nanoparticles injection, the mice were sacrificed. The spleen is the largest secondary lymphoid organ in the body, rich in immune cells, and it is the main site of the immune response. The change in spleen coefficient is an early and intuitive manifestation of the activation or suppression of the body’s immune system. The results showed that the spleen coefficient of mice was almost unchanged before and after NPs injection, indicating that the NPs did not affect the immune system of the mice (Figure 4A). Changes in the levels of inflammatory factors, including IL-6 (Interleukin-6), IL-1β (Interleukin-1β), and TNF-α (Tumor Necrosis Factor-α), in mice are key indicators of their immune status and inflammatory response. These cytokines are mainly produced by activated immune cells or tissue cells, and their elevation or decrease reflects the degree of activation of the inflammatory response, the state of the immune system, and possible pathological damage in mice. There was no significant change in the transcription levels of inflammatory factors in the serum of mice before and after NPs injection, indicating that nanoparticles did not cause inflammatory responses in mice (Figure 4B). The amount of alanine aminotransferase (ALT) and aspartate aminotransferase (AST) in serum did not change after the injection of NPs, indicating that the nanoparticles were not hepatotoxic (Figure 4C). The results of the blood routine test showed that the NPs did not affect the number of red blood cells, white blood cells, neutrophils, monocytes, lymphocytes, and other cells, indicating that the NPs had good blood compatibility (Figure 4D). Tissue sections of mice showed that the NPs had no toxicity to the heart, liver, spleen, lung, and kidney (Figure 4E). In conclusion, the NPs had no obvious toxicity to the immune system, blood, and organs of mice.

### 3.4. NPs/6-Shogaol Induced the Apoptosis of Colon-26 Cells In Vitro

**Figure 5:**
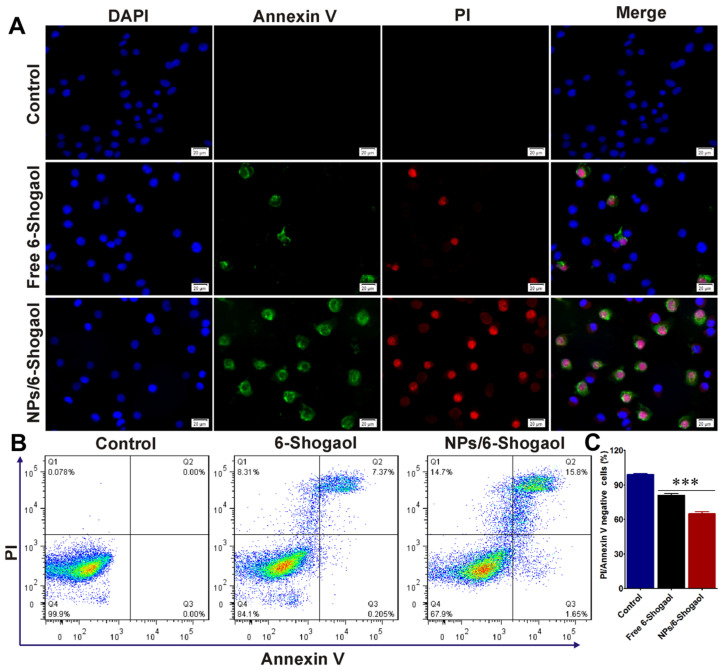
**NPs/6-Shogaol Induced Apoptosis in HT-29 Cells In Vitro.** (**A**) Representative fluorescence images of Annexin V-FITC/PI co-stained HT-29 cells treated with free 6-shogaol or NPs/6-shogaol. Scale bar: 20 µm. (**B**) Apoptosis analysis by flow cytometry. (**C**) Quantitative analysis of Annexin V-FITC/PI-positive apoptotic cells from panel B. Data are representative of three independent experiments. *** *p* < 0.001.

6-Shogaol, the primary active component of dried ginger, induces apoptosis through multiple pathways. To choose the concentration of 6-shogaol, before the cell apoptosis study, the selection of optimum dose was actually performed. When the concentration of 6-shogaol was higher than 5 µg/mL, the cell Viability of HT-29 cells was significantly decreased (Figure S5). Therefore, we chose 5 µg/mL of 6-shogaol for the following study. In this study, 6-shogaol was encapsulated into albumin nanoparticles (designated as NPs/6-shogaol). Colon-26 cells were treated with either free 6-shogaol or NPs/6-shogaol, and apoptosis was evaluated by Annexin V-FITC/PI co-staining in both HT-29 and Colon-26 cells. Representative fluorescence images revealed markedly stronger red fluorescence in the NPs/6-shogaol-treated groups compared to controls, indicating a significantly higher proportion of dead cells (Figure 5A and Figure S6A). The cells in the free 6-shogaol treating group also died, but the number of dead cells was far less than that in the NPs/6-shogaol treating group (Figure 5A and Figure S6A). Next, flow cytometry analysis using Annexin V-FITC and propidium iodide (PI) staining revealed a significant increase in apoptotic cells following treatment with the NPs/6-shogaol or free 6-shogaol, compared with the untreated control group in both HT-29 cells and Colon-26 cells. The cells localized in the Annexin V-FITC^−^/PI^−^ quadrant (lower left) represented viable, non-apoptotic cells. The cells appeared in the Annexin V-FITC^+^/PI^−^ quadrant (lower right), indicative of early apoptosis, while the late apoptotic/necrotic quadrants (Annexin V^+^/PI^+^ and Annexin V^−^/PI^+^) cells were observed in the upper right. The combined total apoptotic fraction (early plus late apoptotic cells) was significantly elevated in the NPs/6-shogaol treated group versus the control group, which demonstrated a clear pro-apoptotic effect of the NPs/6-shogaol (Figure 5B,C and Figure S6B,C). In conclusion, loading 6-shogaol into albumin nanoparticles can effectively promote cell death through apoptosis, while the effect of free 6-shogaol was not as good as loading it into nanoparticles, which may be because nanoparticles can promote the amount of drug into cells.

### 3.5. NPs/6-Shogaol Have In Vivo Therapeutic Efficacy in HT-29 Tumor-Bearing Mice

**Figure 6:**
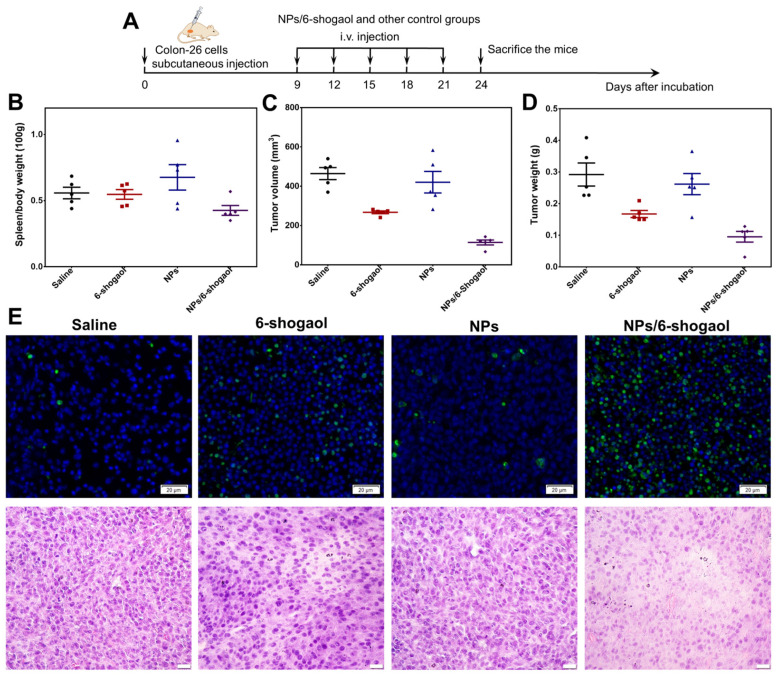
**NPs/6-Shogaol Exhibits In Vivo Therapeutic Efficacy in HT-29 Tumor-Bearing Mice.** (**A**) Schematic of the experimental design. HT-29 tumor-bearing mice were randomly allocated into four groups: control, free 6-shogaol, blank NPs, and NPs/6-shogaol. Corresponding treatments were administered intravenously on days 9, 12, 15, 18, and 21 post-tumor inoculation (n = 5). (**B**) Spleen index calculated at the endpoint (n = 5). (**C**) Quantification of tumor volumes across groups at the end of the study. Data are shown as mean ± SD (n = 5). (**D**) Tumor weights measured at the experimental endpoint (n = 5). (**E**) Apoptosis in HT-29 tumor tissues was assessed by TUNEL staining (green signal). Scale bar: 20 µm. Representative H&E-stained tumor sections are also shown. Scale bar: 20 µm.

In order to verify the therapeutic effect of the NPs/6-shogaol on colon cancer in vivo, the subcutaneous xenograft tumor model was established with HT-29 cells and treated with the indicated drugs (Figure 6A). The results of the spleen coefficient of mice in each group were almost identical, indicating that the treatment did not affect the immune system of the mice (Figure 6B). After the treatment, the mice were sacrificed, and the subcutaneous tumors were removed. The statistical results of the tumor volume and weight indicated that the treatment with the NPs alone had little effect on the growth of the tumors (Figure 6C,D). Free 6-shogaol had a certain inhibitory effect on the tumor growth, while the NPs/6-shogaol formed by loading 6-shogaol into the NPs could effectively inhibit the growth of the tumors in mice (Figure 6C,D). TUNEL is a commonly used assay for detecting cell apoptosis, which detects apoptotic cells by exploiting the terminal deoxynucleotidyl transferase (TdT) enzyme to catalyze the addition of fluorescently or enzymatically tagged dUTP nucleotides specifically onto the exposed 3′-hydroxyl ends of DNA fragments generated during apoptosis-specific DNA fragmentation, enabling their visual detection. The results showed that in the control group and the NPs group, there were almost no cells labeled with green fluorescence. However, in the free 6-shogaol treatment group, there was a certain amount of green fluorescence, while in the NPs/6-shogaol treatment group, a large amount of green fluorescence was observed in the cells, indicating that the NPs/6-shogaol treatment can effectively activate cell apoptosis (Figure 6E). Furthermore, hematoxylin-eosin (H&E) staining was also used to observe the overall morphological structure of the tumor cells. The results showed that in the control group, the cell morphology was irregular, the cell nucleus was significantly enlarged, the ratio of nucleus to cytoplasm was unbalanced, and the cell division was abnormal. However, in the NPs/6-shogaol treatment group, the cell size was relatively uniform, the cell nucleus was round or oval in shape, and the morphology was relatively consistent, and there was less cell division, which indicated that NPs/6-shogaol can effectively inhibit the division of tumor cells (Figure 6E). H&E staining of major organs revealed no histopathological alterations in the heart, liver, spleen, lungs, or kidneys of mice across all treatment groups: free 6-shogaol, blank NPs alone, or NPs/6-shogaol (6-shogaol-loaded nanoparticles) (Figure S7). In conclusion, NPs/6-shogaol can effectively inhibit the growth of subcutaneous HT-29 tumors in mice, and this might be achieved by activating apoptosis to kill tumor cells.

### 3.6. Neutrophil Elastase (NE) Expression in HT-29 Tumor-Bearing Mice

**Figure 7:**
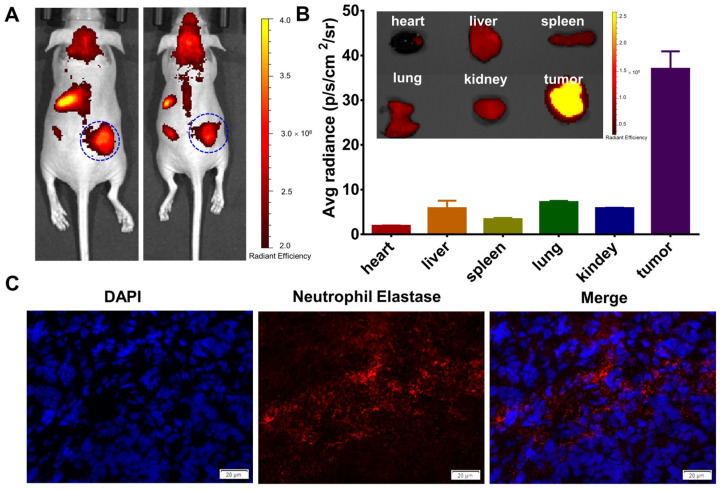
**Neutrophil elastase (NE) expression in HT-29 tumor-bearing mice.** (**A**) The in vivo images of HT-29 tumor-bearing mice traced by the neutrophil elastase 640 FAST imaging agent. (**B**) Ex vivo organ plots and fluorescence intensity statistics of HT-29 tumor-bearing mice traced by neutrophil elastase. (**C**) Immunofluorescent staining of NE on colon cancer tissue, Scale bar: 20 μm.

NE is a serine protease secreted by neutrophils, which is involved in inflammatory response, pathogen clearance, and tissue remodeling, and is extremely active in a variety of inflammatory diseases. NE 680 FAST imaging reagent is a molecular probe for the specific detection of NE activity, which is mainly used for real-time, high-sensitivity imaging in medical research. First, the subcutaneous xenograft tumor model was established with HT-29 cells. The results of in vivo imaging showed that NE was significantly expressed at the tumor site (Figure 7A). Fluorescence imaging of isolated organs showed that NE was little accumulated in the heart, liver, spleen, lung, and kidney, but was significantly accumulated at the tumor site (Figure 7B). Immunofluorescent staining also showed the high expression of NE in colon cancer (Figure 7C). NAS-DCE staining is a cytochemical staining method specific for the granulocyte system, which can indirectly reflect the expression of NE. The results showed that there was a large amount of NE expression in the tumor cells (Figure S8). In conclusion, NE was significantly expressed in the tumor cells of HT-29 tumor-bearing mice.

### 3.7. NPs/6-Shogaol Inhibited the Proliferation of Tumor Cells by Activating Apoptosis

**Figure 8:**
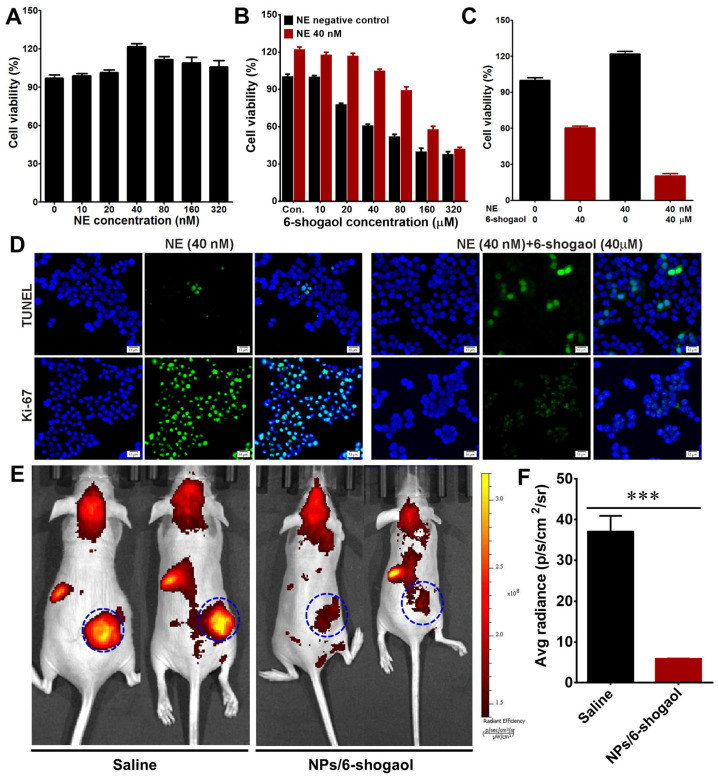
**NPs/6-Shogaol Suppresses Tumor Cell Proliferation by Inducing Apoptosis.** (**A**) Viability of HT-29 cells treated with varying concentrations of neutrophil elastase (NE). (**B**) Viability of HT-29 cells exposed to different concentrations of 6-shogaol, with or without 40 nM NE. (**C**) Viability of HT-29 cells under the indicated treatment conditions. (**D**) Apoptosis in HT-29 tumors was evaluated by TUNEL staining (green). Cell proliferation was assessed via Ki-67 immunofluorescence staining. Scale bar: 10 μm. (**E**) In vivo fluorescence imaging of HT-29 tumor-bearing mice using the neutrophil elastase 680 FAST imaging probe. (**F**) Quantitative analysis of fluorescence intensity in tumors detected with the neutrophil elastase probe. *** *p* < 0.001.

Under the treatment with different concentrations of NE, the cell viability all increased, and the cell viability of HT-29 cells was the highest at the 40 nM concentration, which indicated that NE can, to a certain extent, promote the growth of tumor cells (Figure 8A). The cell viability of the cells treated with NE was all higher than that of the untreated groups, while the cell viability decreased as the concentration of 6-shogaol increased, whether with 40 nM NE or not. And at a concentration of 320 nM, the cell viability of both was approximately the same (Figure 8B). Under the treatment condition of 40 nM NE, the cell survival rate was as high as 120%, but after being exposed to 40 nM of the 6-shogaol, the cell viability dropped to below 30% (Figure 8C). The proportion of actively proliferating cells in the tumor is evaluated by identifying the Ki-67 protein in the cell nucleus. The results showed that in the NE treatment group, the number of cells labeled with green fluorescence significantly increased. However, after the addition of 6-shogaol, the green fluorescence disappeared significantly, indicating that NE treatment activated the proliferation of tumor cells, while 6-shogaol effectively inhibited the proliferation of tumor cells (Figure 8D). After the addition of 6-shogaol, the amount of cell apoptosis significantly increased, indicating that 6-shogaol inhibited the proliferation of tumor cells by activating apoptosis (Figure 8D). After administering drug NPs/6-shogaol to HT-29 tumor-bearing mice, the expression level of NE significantly decreased, suggesting that NPs/6-shogaol can effectively inhibit the expression of NE at the tumor site (Figure 8E,F). In conclusion, NE can promote the proliferation of tumor cells, while NPs/6-shogaol inhibited the proliferation of tumor cells by activating apoptosis, thereby achieving the therapeutic effect on tumors.

### 3.8. NPs/6-Shogaol Have In Vivo Therapeutic Efficacy in AOM/DSS-Induced Orthotopic Colorectal Cancer Model in Mice

**Figure 9:**
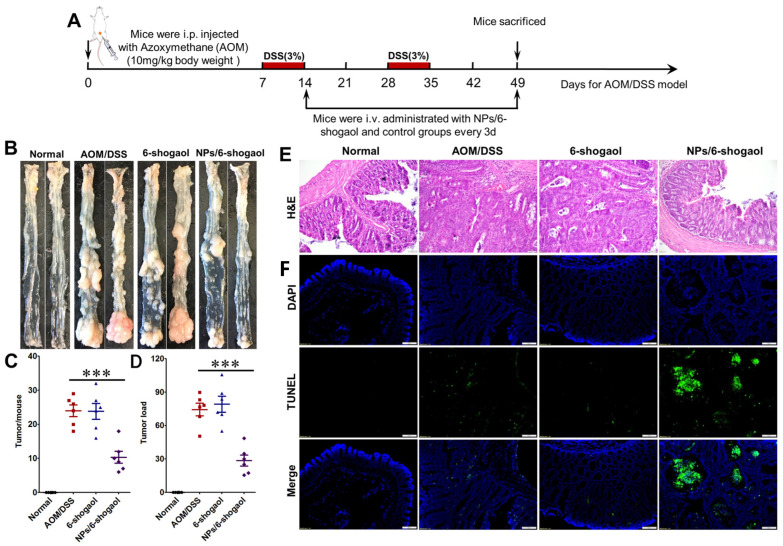
**NPs/6-Shogaol Exhibits In Vivo Efficacy in an AOM/DSS-Induced Orthotopic Colorectal Cancer Mouse Model.** (**A**) Schematic illustrating the establishment of the orthotopic colon cancer model using azoxymethane (AOM) and dextran sodium sulfate (DSS). (**B**) Representative macroscopic images of isolated colons from each treatment group. (**C**) Quantification of tumor number per colon (n = 5). (**D**) Assessment of tumor burden per mouse (n = 5). (**E**,**F**) Histological evaluation of tumors by H&E staining (**E**) and apoptosis detection by TUNEL staining (**F**). Scale bar = 50 μm. *** *p* < 0.001.

In order to further verify the therapeutic effect of NPs/6-shogaol on colorectal cancer, the orthotopic colorectal cancer model induced by AOM/DSS was established, and the mice were randomly divided into four groups (control group, AOM/DSS group, 6-shogaol group, and NPs/6-shogaol group) (Figure 9A). At the end of treatment, mice were euthanized. Dissected colons revealed that in the control group, the colonic wall was soft and pliable with smooth, translucent serosa; the mucosa was intact, the lumen was patent, and no tumor structures were observed (Figure 9B). In contrast, the AOM/DSS group exhibited thickened colonic walls, roughened serosal surfaces with multiple porcelain-white tumor nodules, mucosal ulcerations, luminal stenosis, and multifocal elevated tumors, indicating significantly increased tumor load (Figure 9B–D). In the treatment group (NP/6-shogaol), restoration of colonic wall integrity and mucosal morphology was noted, accompanied by a marked reduction in tumor number and overall tumor load (Figure 9B–D). The results of H&E staining of mouse colonic tissue revealed that, compared to the control group exhibiting well-organized architecture, intact mucosa, abundant goblet cells, and minimal inflammatory infiltrate, the AOM/DSS group displayed crypt distortion, depletion of goblet cells, irregular cellular morphology, and marked inflammatory infiltration. Following NPs/6-shogaol administration, amelioration of various pathological features was observed in the colon, demonstrating the significant therapeutic efficacy of NPs/6-shogaol against colorectal cancer (Figure 9E). TUNEL staining results demonstrated that a negligible green fluorescent signal was detectable in the control group, the AOM/DSS group, and the group receiving free 6-shogaol. In contrast, administration of 6-shogaol encapsulated within nanoparticles (NPs/6-shogaol) induced intense green fluorescence within the cells, indicating that NPs/6-shogaol effectively induces apoptosis in tumor cells (Figure 9F). In summary, NPs/6-shogaol demonstrated significant therapeutic efficacy against AOM/DSS-induced orthotopic colon cancer in mice, likely mediated by the induction of apoptosis to eliminate cancer cells.

## 4. Discussion

Colorectal cancer (CRC) remains a formidable clinical challenge, largely due to the inefficient penetration of chemotherapeutics across the blood-tumor barrier and the associated systemic toxicities [29]. In this study, we developed a neutrophil-mediated albumin nanoplatform for the targeted delivery of 6-shogaol, a natural anti-tumor compound derived from ginger, to colon tumors. Our findings demonstrate that this bioinspired delivery strategy significantly enhances therapeutic efficacy while minimizing off-target effects, offering a promising approach to overcome current limitations in CRC treatment.

The rationale for employing neutrophils as cellular carriers stems from their innate ability to migrate toward inflammatory and tumor microenvironments [30]. Activated neutrophils exhibit a high affinity for albumin nanoparticles, enabling effective “hitchhiking” and subsequent transmigration across vascular barriers. Our results confirm that DiL-labeled albumin NPs were efficiently internalized by Ly-6G/Ly-6C^+^ neutrophils in tumor-bearing mice, facilitating targeted accumulation in both subcutaneous and orthotopic tumor models. This aligns with emerging evidence that immune cells can serve as Trojan horses for nanoparticle delivery, leveraging their biological mobility and immune-evasion capabilities [31].

The formulated NPs/6-shogaol exhibited favorable physicochemical properties, including a uniform nano-scale size (~150 nm), negative zeta potential, and high drug loading efficiency. In vitro studies revealed excellent biocompatibility, with no significant cytotoxicity or apoptosis induction in Colon-26 and HT-29 cell lines, even at high nanoparticle concentrations. Importantly, NPs/6-shogaol demonstrated enhanced pro-apoptotic activity compared to free 6-shogaol, likely attributable to improved cellular uptake and sustained drug release mediated by the albumin carrier.

In vivo therapeutic evaluations further underscored the superiority of the neutrophil-hitchhiking system. In HT-29 subcutaneous xenograft models, NPs/6-shogaol treatment resulted in significant tumor regression, accompanied by elevated TUNEL-positive apoptotic cells and restored tissue morphology. Notably, this anti-tumor effect was achieved without eliciting systemic inflammation or organ toxicity, as evidenced by stable cytokine levels, normal liver enzyme activities, and unaltered hematological parameters. These findings highlight the favorable safety profile of our nanoplatform, a critical consideration for translational applications [32,33].

Mechanistically, we identified neutrophil elastase (NE) as a key molecular target in the tumor microenvironment. NE, overexpressed in CRC tissues, has been implicated in tumor progression and metastasis [34]. Our data show that 6-shogaol effectively suppresses NE expression and activity, thereby inhibiting tumor cell proliferation and inducing apoptosis. This dual functionality—targeted delivery combined with NE modulation—represents a novel therapeutic axis that extends beyond conventional cytotoxic approaches.

The therapeutic potential of NPs/6-shogaol was further validated in a clinically relevant AOM/DSS-induced orthotopic CRC model. Administration of NPs/6-shogaol markedly reduced tumor burden, restored colonic architecture, and enhanced apoptosis in neoplastic lesions, outperforming both free drug and blank nanoparticle controls. These results reinforce the translational relevance of our strategy for treating advanced and invasive forms of CRC.

## 5. Conclusions

In conclusion, this study presents a neutrophil-driven albumin nanoparticle system for the targeted delivery of 6-shogaol to colon tumors. By harnessing the migratory capacity of neutrophils and the tumor-suppressive activity of 6-shogaol, our platform achieves enhanced drug accumulation, potent anti-tumor efficacy, and minimal systemic toxicity. Future studies may explore the applicability of this approach to other inflammation-associated cancers, as well as the potential for combination with immunotherapeutic agents. Overall, this work contributes to the growing field of cell-mediated drug delivery and offers a promising strategy for improving outcomes in CRC therapy.

## Data Availability

All the other data supporting the findings of this study are available within the article and from the corresponding author upon reasonable request.

## Supplementary Materials

The following supporting information can be downloaded at: https://www.mdpi.com/article/10.3390/jfb17010036/s1, Figure S1: Characterization of NPs/6-shogaol; Figure S2: 6-Shogaol encapsulated in albumin NPs was quantified by HPLC; Figure S3: Effects of albumin NPs on apoptosis of Colon-26 cells and HT-29 cells were assessed; Figure S4: Hemolysis assay of albumin NPs to blood cells were performed; Figure S5. The selection of the optimum dose for 6-shogaol. Figure S6: NPs/6-shogaol induced the apoptosis of Colon-26 cells in vitro; Figure S7: H&E staining for vital organs from HT-29 tumor-bearing mice; Figure S8: Leder Stain evaluated the expression of Neutrophil elastase (NE) in colon cancer tissues.
