# Supplementary material for: Albumin Nanoparticles Harness Activated Neutrophils to Cross Vascular Barriers for Targeted Subcutaneous and Orthotopic Colon Cancer Therapy
Source: J Funct Biomater. 2026 Jan 10;17(1):36. doi: 10.3390/jfb17010036 (PMC12842495; doi:10.3390/jfb17010036)
Supplement: Supplementary file 1 [file jfb-17-00036-s001.zip › jfb-4060090-supplementary.pdf]

Supporting Information

Materials

Bovine serum albumin (No: A2153), AST activity assay kit (No: MAK055), ALT activity assay kit (No: MAK052), In situ cell death detection kit (No: 11684795910), Naphthol AS-D Chloroacetate (Specific Esterase) Kit (No: 91C-1KT), 6-shogaol (No: SMB00311) and analytical standard (No: 39303) were purchased from Sigma-Aldrich (St. Louis, MO, USA). Alexa Fluor® 488 anti-mouse Ly-6G/Ly-6C (Gr-1) antibody (No: 108417) was from Biolegend (San Diego, CA, USA). ATP Determination Kit (No: A22066), MTT (3-(4,5-Dimethylthiazol-2-yl)-2,5-Diphenyltetrazolium Bromide) (No: 6494), LIVE/DEAD® Cell Imaging Kit (488/570) (No: R37601) and Annexin V-FITC/propidium iodide (PI) apoptosis detection kit (No: V13245) were obtained from ThermoFisher Scientific (Eugene, OR, USA). Duoset enzyme-linked immune sorbent assay (ELISA) kits for IL-6, IL-1β, and TNF-α were purchased from R&D Systems (Minneapolis, MN, USA). Neutrophil Elastase 680 FAST™ (No: NEV11169) was from PerkinElmer (Waltham, MA, USA). Neutrophil Elastase Antibody (N-18) (No: sc-9518) was from Santa Cruz Biotechnology (Santa Cruz, CA, USA). Natural human Neutrophil Elastase protein (No: ab91099) and Anti-ki67 antibody (No: ab16667) were from Abcam (Cambridge, MA, USA). 1, 1'-dioctadecyl-3,3,3',3'-tetramethylindocarbocyanine perchlorate (DiL) was purchased from Promokine (Heidelberg, Germany).

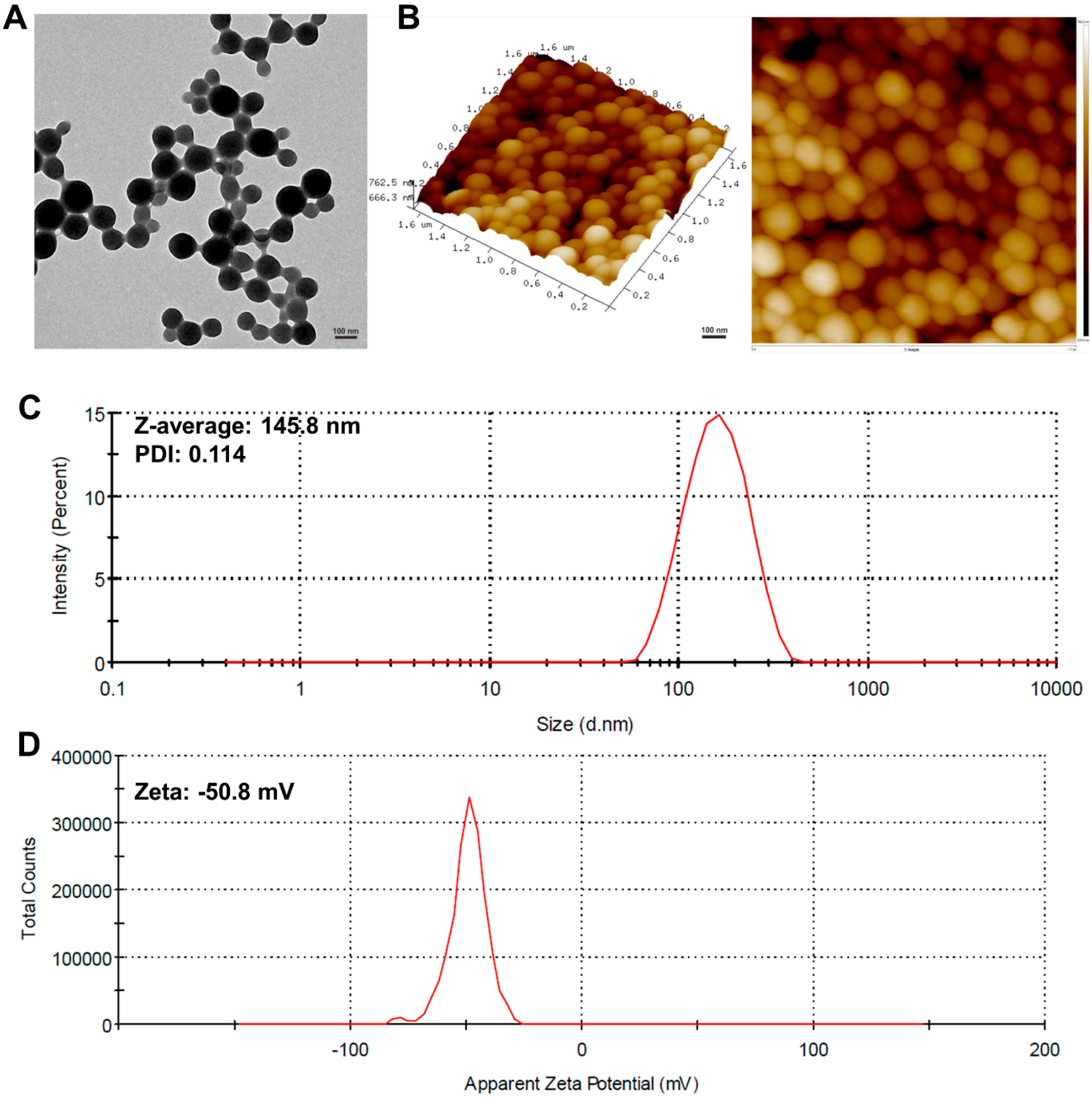

Figure S1. Characterization of NPs/6-shogaol. A. Representative Transmission Electron Microscope (TEM) image of NPs/6-shogaol. **B.** Representative Atomic Force Microscope (AFM) image of NPs/6-shogaol. **C-D.** Particle size and zeta potential of NPs/6-shogaol were characterized by dynamic light scattering (DLS) (n = 3).

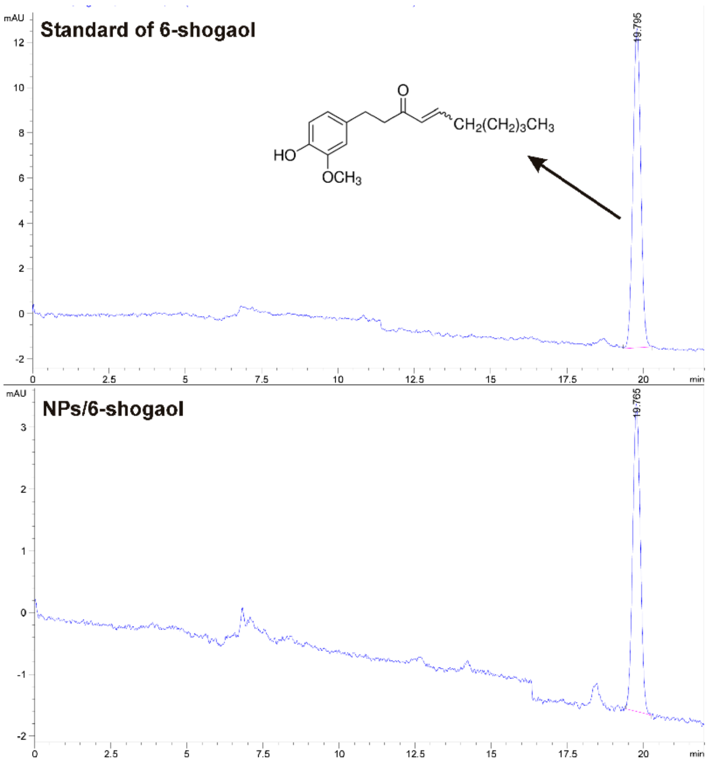

Figure S2. 6-Shogaol encapsulated in albumin NPs was quantified by HPLC. The detection wavelength was set at 282 nm.

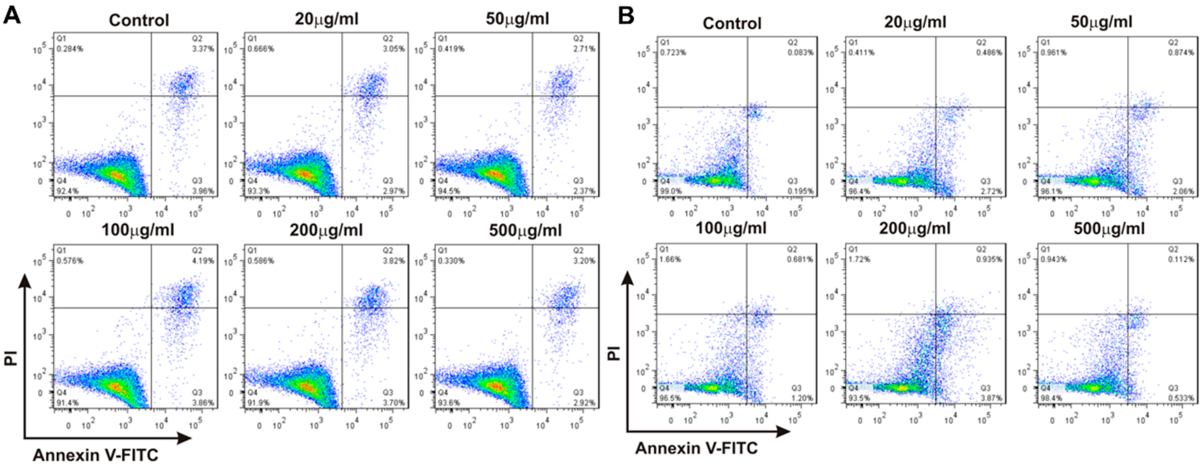

Figure S3. The effects of albumin NPs on the apoptosis of Colon-26 cells and HT-29 cells were assessed. **A.** Colon-26 cells were incubated with albumin NPs in different concentrations, and the apoptotic state of cells was evaluated by flow cytometry with Annexin V-FITC/PI staining. **B.** HT-29 cells were incubated with albumin NPs in different concentrations, and the apoptotic state cells were evaluated by flow cytometry with Annexin V-FITC/PI staining.

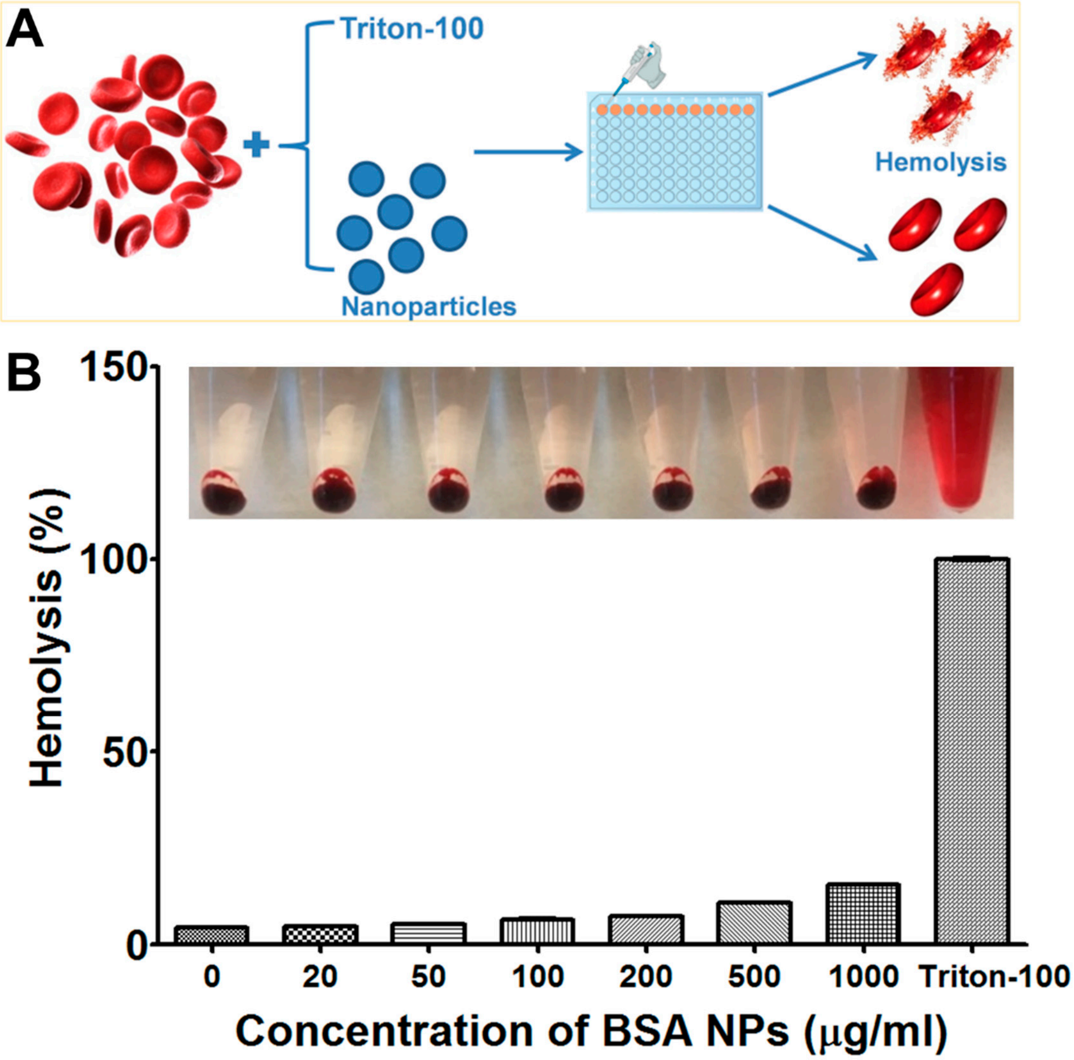

Figure S4. Hemolysis assay of albumin NPs on blood cells was performed. A. Schematic of hemolysis. **B.** Degree of hemolysis triggered by different concentrations of albumin NPs (20, 50, 100, 200, 500, and 1000 μg/ml), 1% Triton-100 used as a positive control (n = 3).

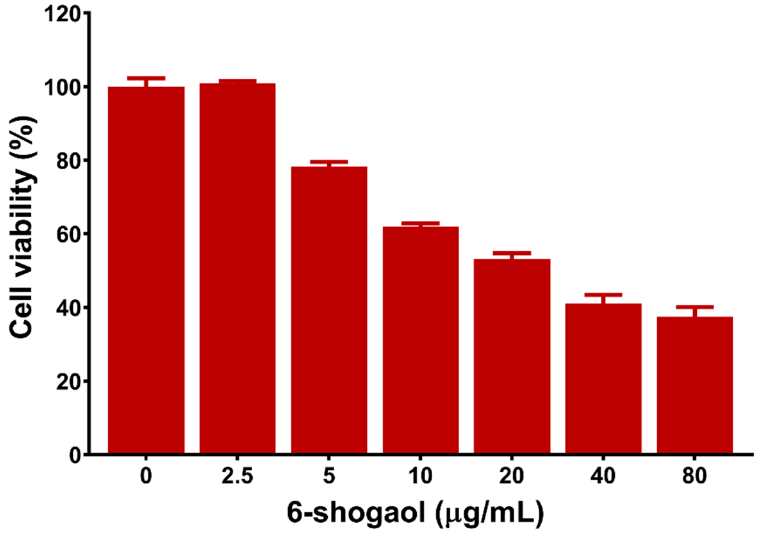

Figure S5. The selection of the optimum dose for 6-shogaol.

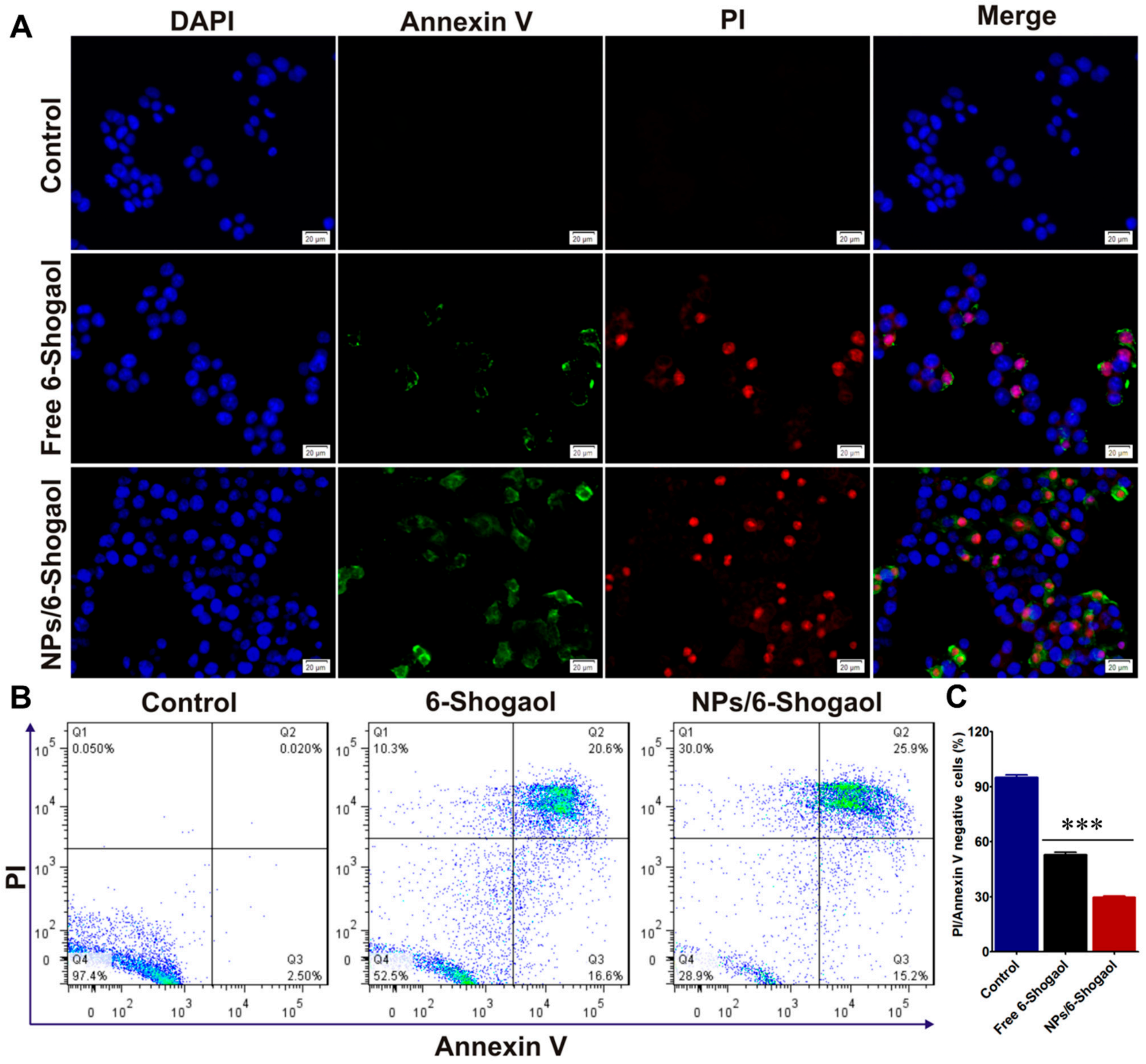

Figure S6. NPs/6-shogaol induced the apoptosis of colon-26 cells *in vitro*. A. Colon-26 cells were treated with free 6-shogaol and NPs/6-shogaol, and representative fluorescence images of Colon-26 cells co-stained with Annexin V-FTIC and PI were captured. Scale bar: 20 μm. **B.** Apoptosis was measured by flow cytometry. **C.** Quantification of the Annexin V-FTIC/PI-positive apoptotic cells shown in panel B. The data are representative of three independent experiments. ***p<0.001.

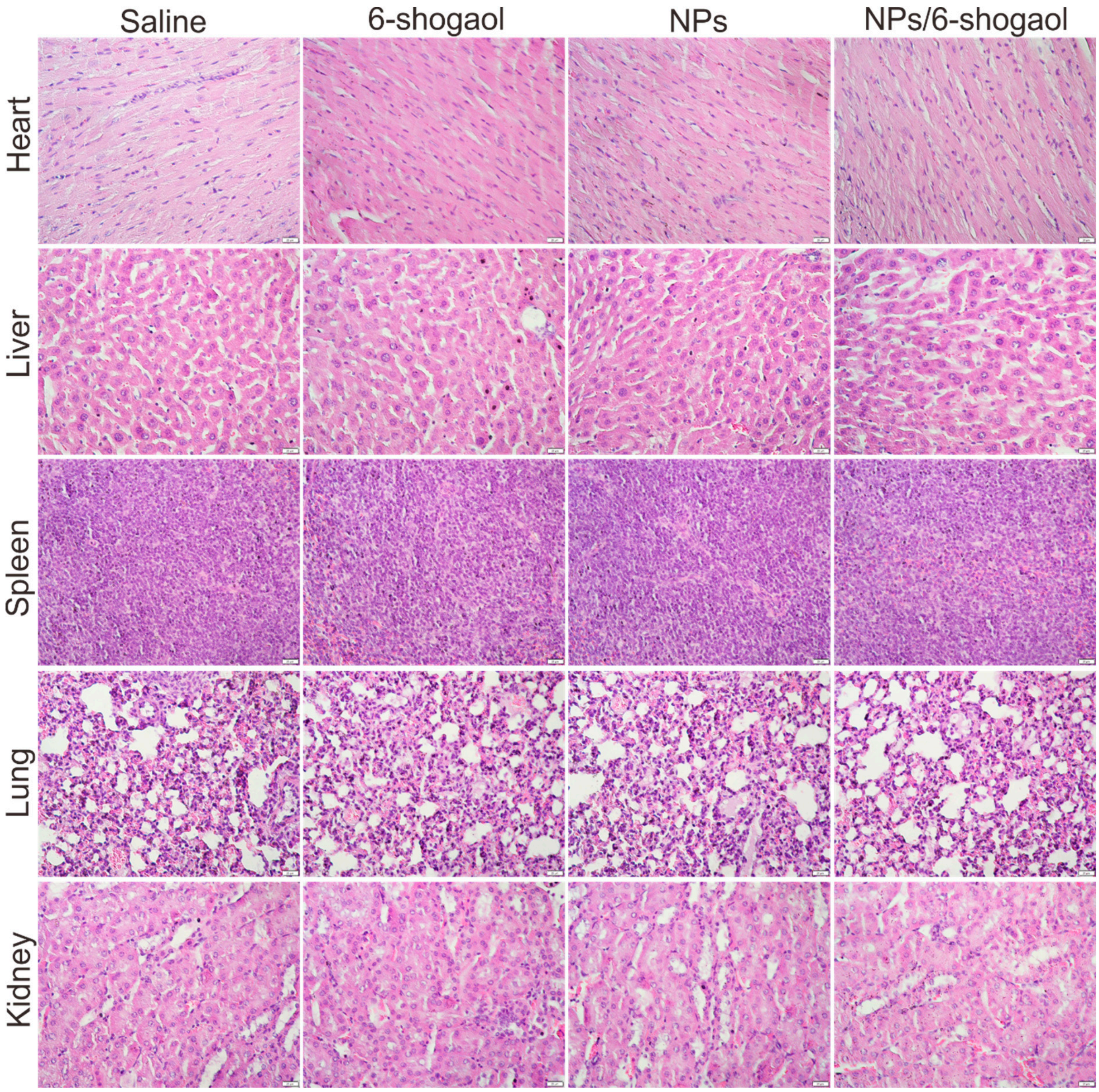

Figure S7. H&E staining for vital organs from HT-29 tumor-bearing mice. HT-29 tumor-bearing mice were treated with saline, free 6-shogaol, albumin NPs, and NPs/6-shogaol. The vital organs from each group were subjected to H&E histologic examination. Scale bar: 20 μm.

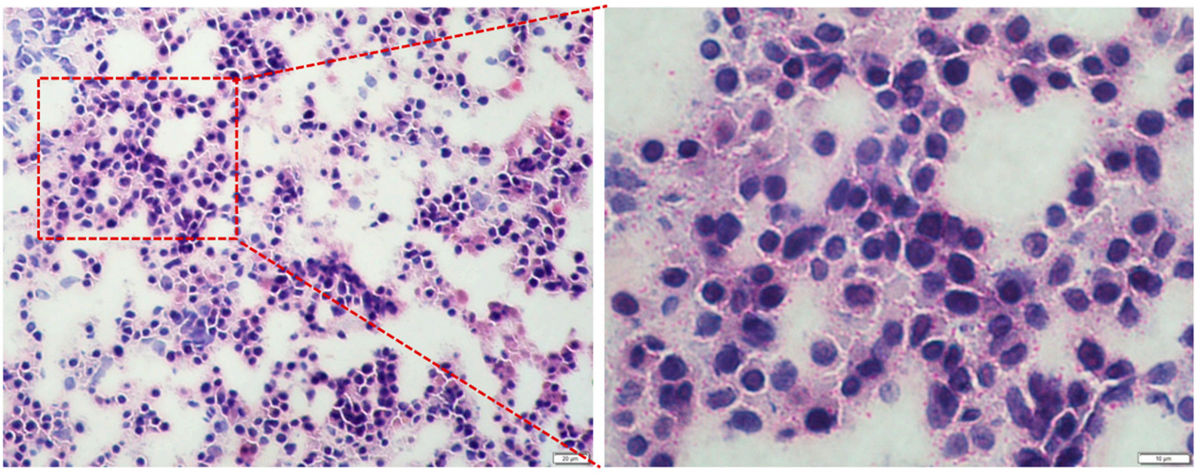

Figure S8. Leder Stain evaluated the expression of Neutrophil Elastase (NE) in colon cancer tissues. Naphthol AS-D Chloroacetate (Specific Esterase) Kit was used for Leder Stain; Naphthol AS-D chloroacetate can be hydrolyzed into Naphthol AS-D by esterase to mix with diazonium salt and form an insoluble red-brown precipitate in the cytoplasm.
